# Robust Localization of Maneuvering UAV Swarms Using Lateral Doppler Resolution

**DOI:** 10.3390/s26196121

**Published:** 2026-09-26

**Authors:** Chenfei Yang, Zhuangzhi Han, Jinwei Jia, Limin Liu, Lin Shi

**Affiliations:** Shijiazhuang Campus, Army Engineering University of PLA, Shijiazhuang 050003, China; chenfeiyang@aeu.edu.cn (C.Y.); serverus@aeu.edu.cn (J.J.); yhj0708@aeu.edu.cn (L.S.)

**Keywords:** UAV swarms, swarm target resolution, lateral Doppler resolution, backprojection algorithm

## Abstract

A radar with a limited aperture may lack sufficient cross-range resolution to separate closely spaced members of an unmanned aerial vehicle (UAV) swarm. This paper proposes a localization method that combines lateral-Doppler scaling, data-driven estimation of the equivalent cross-range reference velocity, and acceleration-hypothesis backprojection (BP). The reference velocity is estimated by maximizing a regularized spatial-concentration criterion, and target-dependent motion is accommodated by pixel-wise maximum fusion over an acceleration grid. For a fixed 15-target formation with acceleration scales from 1.8 to 9 m/s^2^, the method achieved conditional localization RMSE values of 0.81–1.15 m and single-scene detection rates of 0.87–1.00. The differences relative to range–Doppler (RD) and second- and third-order Taylor-Chirplet processing were most pronounced at the larger acceleration scales. Monte Carlo experiments with randomized noise and Swerling-I scattering further showed that maximum fusion yielded higher matched-detection probabilities and lower miss-penalized RMS errors than mean fusion and a zero-acceleration baseline over per-pulse SNRs from −18 to −6 dB. Acceleration-grid, PRF, and reference-velocity sweeps characterized the associated computational trade-offs and operating range.

## 1. Introduction

Dense UAV swarms are challenging radar targets because nearby members can occupy similar range–angle cells and exhibit similar motion. Long-range surveillance nevertheless requires estimates of both target count and individual position [1,2,3]. Conventional radars may therefore detect a formation as a whole while failing to separate its closely spaced members.

The lateral resolution of a conventional radar system is limited by antenna aperture and detection range. Radar must estimate a swarm’s size and spatial distribution early enough to support an air-defense response [4], a requirement that can exceed the Rayleigh limit of a real-aperture radar. Existing studies have examined multidimensional joint resolution [5], super-resolution direction-of-arrival (DOA) estimation [6,7,8], and multiple-input multiple-output (MIMO) virtual apertures [9,10,11,12]. Their long-range angular resolution remains constrained when platform size limits the total array aperture [13]. Lateral-Doppler resolution follows a different principle: sub-targets at different cross-range positions produce different Doppler histories as the viewing aspect changes. The resulting cross-range resolution depends on wavelength and accumulated aspect angle rather than directly on target range [14,15]. Practical use requires reliable compensation of bulk translational motion over the coherent aperture. Inverse synthetic aperture radar (ISAR) is one implementation of this principle [16].

Conventional ISAR is difficult to apply to slow-moving UAV swarms. At long range, an adequate aspect change may require a long integration interval, which also magnifies relative motion among members of a maneuvering formation. Their Doppler histories can then overlap within the same range cell, degrading both separation and localization. The authors previously proposed a lateral-Doppler localization method for rigid UAV formations [17]. Extending that formulation to a maneuvering swarm raises two issues. First, one motion history cannot represent members with different effective accelerations, while estimating a separate high-order model for every member is poorly constrained. Second, the earlier formulation requires a rotation-rate estimate to map Doppler frequency to cross-range position, which is generally unavailable for a non-cooperative swarm.

This paper combines lateral-Doppler scaling and BP with two components tailored to target-dependent motion. A regularized spatial-concentration criterion estimates an equivalent cross-range reference velocity from the echo data, so no external rotation-rate input is required. Acceleration-hypothesis BP then forms a bank of spatial images and performs pixel-wise hypothesis selection. The fused image is processed by two-dimensional detection and sub-grid position refinement. Fixed-formation comparisons with RD and second- and third-order Taylor-Chirplet processing are supplemented by Monte Carlo sensitivity experiments for acceleration-grid density, fusion strategy, SNR, Swerling-I scattering, PRF, and reference-velocity mismatch.

## 2. Swarm Target Echo Model and Problem Analysis

The radar is assumed to operate in staring mode and illuminate the target area with coherent pulses. The following model describes the resulting echo and the limitations of conventional processing under target-dependent motion.

### 2.1. Swarm Target Echo Model Construction

The observation geometry is shown in Figure 1. The radar is treated as a fixed station, and the local coordinate system is centered at the swarm reference point O. The *y*-axis is the range direction and the *x*-axis is the cross-range direction. Coarse envelope alignment and phase correction remove the common radial translation of O before the proposed imaging stage. The remaining model describes motion relative to that compensated reference.

Let $x_{i}$ and $y_{i}$ be the initial cross-range and range offsets of sub-target *i*, respectively. Let $v_{\mathrm{ref}}$ be the common equivalent cross-range reference velocity generated by the change in viewing aspect, and let $a_{i}$ be the target-dependent effective cross-range acceleration. The cross-range trajectory and exact slant range are

(1) $$\begin{array}{cc} x_{i}\left(t_{m}\right) & =x_{i}+v_{\mathrm{ref}}t_{m}+\frac{1}{2}a_{i}t_{m}^{2},\\ R_{i}\left(t_{m}\right) & =\sqrt{{\left(R_{\mathrm{ref}}+y_{i}\right)}^{2}+x_{i}^{2}\left(t_{m}\right)},\;\;i=1,2,\ldots ,N_{t} \end{array}$$

where $N_{t}$ is the number of sub-targets and $R_{\mathrm{ref}}$ is the reference range. The corresponding slant-range deviation used in the phase history is

(2) $$\Delta R_{i}\left(t_{m}\right)=R_{i}\left(t_{m}\right)-R_{\mathrm{ref}}$$

Under the far-field approximation, an aspect rate ω corresponds to $v_{\mathrm{ref}}≃\omega R_{\mathrm{ref}}$, so $v_{\mathrm{ref}}$ has units of m/s. It is distinct from radial translation and is not estimated separately for every target. The proposed processor assumes the second-order cross-range trajectory in Equation (1); higher-order target-dependent motion lies outside the present model. The residual-phase analysis below concerns Taylor truncation of this stated trajectory.

Assume that the radar transmits a linear frequency-modulated signal. After demodulation, the range-frequency echo from the *i*-th target is

(3) $$s_{i}\left(f_{r},t_{m}\right)=A_{i}\cdot rect\left(\frac{f_{r}}{B}\right)\cdot \exp\left[-j\frac{4\pi }{c}\left(f_{c}+f_{r}\right)\Delta R_{i}\left(t_{m}\right)\right]$$

where $f_{r}$ is the range-frequency variable, $t_{m}$ is slow time, $A_{i}$ is the complex scattering amplitude, *B* is the signal bandwidth, $f_{c}$ is the carrier frequency, and *c* is the speed of light.

In the swarm observation scenario, echoes from multiple targets overlap in both range and Doppler. The total echo at the *m*-th pulse is

(4) $$S\left(f_{r},t_{m}\right)=\sum_{i=1}^{N_{t}}A_{i}\cdot rect\left(\frac{f_{r}}{B}\right)\cdot \exp\left[-j\frac{4\pi }{c}\left(f_{c}+f_{r}\right)\Delta R_{i}\left(t_{m}\right)\right]+n\left(f_{r},t_{m}\right)$$

where $n(f_{r},t_{m})$ denotes additive noise. Equation (4) defines the swarm echo model used in this paper. Because $x_{i}$, $y_{i}$, and $a_{i}$ differ among sub-targets, their slant-range histories and migration through range cells (MTRC) are also target dependent. We refer to this position- and target-dependent residual motion as space-variant motion.

After inverse transformation in range frequency and matched filtering, let $s(r,t_{m})$ denote the range-compressed data as a function of range *r* and slow time. The BP interpolation below is applied to this range-domain quantity, rather than directly to the range-frequency data $S(f_{r},t_{m})$.

### 2.2. Failure Analysis of Conventional Methods

The range–Doppler (RD) algorithm usually assumes one dominant sub-target. It extracts that target’s phase history to estimate one set of motion-compensation parameters and applies them to all echoes. A Fourier transform along slow time then provides cross-range resolution. This common-history assumption becomes inaccurate for a non-rigid swarm because the offsets and effective accelerations in Equation (1) are target dependent. The remaining phase differences can broaden focused responses and bias their locations.

Using Equations (2) and (3), the baseband phase of the *i*-th sub-target is

(5) $$\phi_{i}\left(t_{m}\right)=-\frac{4\pi }{\lambda }\Delta R_{i}\left(t_{m}\right)$$

The range delay $\Delta R_{i}$ varies with slow time. MTRC occurs when the excursion of this exact range history exceeds one range-resolution cell. For sub-target *i*, the migration over the coherent processing interval T is

(6) $$\Delta R_{\mathrm{MTRC},i}=\max_{t_{m}\in T}R_{i}\left(t_{m}\right)-\min_{t_{m}\in T}R_{i}\left(t_{m}\right)$$

If $\Delta R_{\mathrm{MTRC},i}$ exceeds the range resolution $\rho_{r}$, the target crosses one or more range cells. Because $a_{i}$ is target dependent, one global correction cannot align all sub-target histories.

Second- and third-order Chirplet decomposition methods can reduce the azimuth bias of maneuvering targets. These methods compensate quadratic and cubic polynomial-phase terms, respectively [18,19]. A cubic phase coefficient is not, by itself, evidence of physical jerk: it can also arise when the exact slant range of a second-order cross-range trajectory is expanded in slow time. Let $R_{c}=R_{\mathrm{ref}}+y$ and write a generic effective cross-range trajectory as $x\left(t\right)=x+Vt+at^{2}/2$. Retaining leading far-field terms gives

(7) $$R\left(t\right)\approx R_{c}+\frac{x^{2}}{2R_{c}}+\alpha t+\beta t^{2}+\gamma t^{3}$$

where the first-, second-, and third-order polynomial coefficients are

(8) $$\alpha =\frac{xV}{R_{c}},\;\;\beta =\frac{V^{2}+xa}{2R_{c}},\;\;\gamma =\frac{Va}{2R_{c}}$$

For a representative sub-target, the slow-time echo after range compression is

(9) $$s_{i}\left(t\right)=A_{i}\;\mathrm{rect}\left(\frac{t}{T_{a}}\right)\exp\left\{-j\frac{4\pi }{\lambda }\left[R_{c}+\frac{x^{2}}{2R_{c}}+\alpha t+\beta t^{2}+\gamma t^{3}\right]\right\}+n\left(t\right)$$

Here, $T_{a}$ is the coherent aperture duration, $A_{i}$ is the complex scattering coefficient defined in the echo model, and n(t) is the corresponding slow-time noise term.

A grid search determines the quadratic coefficient β and cubic coefficient γ. These coefficients compensate the corresponding polynomial-phase terms and improve slow-time focusing. Physical jerk is outside the second-order trajectory assumed in Equation (1); a higher-order motion model would be required if jerk produced a non-negligible residual over the coherent processing interval.

This expansion uses the scene center as the common reference. A target away from that center retains residual phase. Residual phase also remains when the true motion order exceeds the compensation order.

The absolute slant-range error of the third-order Taylor approximation is plotted in Figure 2. At 11 GHz, the maximum absolute range error over the displayed interval is approximately 0.42 mm, corresponding to a phase error of 0.19 rad (10.9°). The associated coherent-power factor is

(10) $$\eta_{c}={\left|\frac{1}{M}\sum_{m=1}^{M}\exp\left(j\;\delta \phi_{m}\right)\right|}^{2}$$

Since $|\delta \phi_{m}|\leq 0.19$ rad over this interval, the sign-independent bound is $\eta_{c}\geq \cos^{2}\left(0.19\right)≃0.964$, corresponding to no more than approximately 3.6% coherent-power reduction over the displayed interval.

The third issue concerns Doppler ambiguity. From Equation (5), the Doppler frequency of a sub-target is

(11) $$f_{d,i}\left(t_{m}\right)=\frac{1}{2\pi }\frac{\partial \phi_{i}}{\partial t_{m}}=-\frac{2}{\lambda }\frac{dR_{i}\left(t_{m}\right)}{dt_{m}}$$

For the compensated second-order model in Equation (1), $dR_{i}/dt_{m}=x_{i}\left(t_{m}\right)\left(v_{\mathrm{ref}}+a_{i}t_{m}\right)/R_{i}\left(t_{m}\right)$. Thus, the residual Doppler is generated by the exact slant-range history; it is not a separately introduced radial-velocity term.

When the radar pulse repetition frequency (PRF) is below the Doppler bandwidth, the observed Doppler frequency is folded:

(12) $$f_{d,i}^{obs}=f_{d,i}-k_{i}\cdot f_{PRF},\;k_{i}\in Z$$

where $k_{i}$ is the ambiguity number, or folding factor, and it may differ among targets. The folded frequency lies in [−PRF/2, PRF/2]. One Doppler cell may therefore contain several sub-targets with different true velocities.

Three mechanisms can contribute to the degradation of RD and second- and third-order Chirplet processing in the tested maneuvering scene. First, a single reference history cannot compensate the target-dependent slant-range histories generated by different positions and effective accelerations. Second, space-variant MTRC and range–Doppler coupling cannot generally be removed by one linear migration correction. Third, when the PRF is below the Doppler bandwidth, different targets may have different ambiguity numbers; mapping folded RD bins directly to cross-range coordinates can then distort the relative swarm geometry.

## 3. Localization Based on Lateral Doppler Resolution

### 3.1. Algorithm Overview

The proposed method combines data-driven cross-range scaling with acceleration-hypothesis BP. Preprocessing first provides coarse estimates of the reference range and equivalent cross-range velocity. The velocity is refined with a regularized spatial-concentration objective. For each acceleration hypothesis, BP evaluates the exact slant-range history on a two-dimensional spatial grid and integrates the phase-compensated samples coherently. Pixel-wise maximum fusion then retains the strongest acceleration-matched response before detection and position refinement. Figure 3 summarizes the processing flow.

Step 1: Perform pulse compression to obtain the range–slow-time data $s(r,t_{m})$. Estimate the reference range $R_{\mathrm{ref}}$ from the slow-time-averaged range-energy profile. Then apply coarse translational compensation using envelope cross-correlation and initial phase correction.

Step 2: Search the candidate velocity grid using the regularized spatial-concentration criterion $J_{\mathrm{reg}}\left(v\right)$. The resulting ${\hat{v}}_{\mathrm{ref}}$ defines the equivalent cross-range scale.

Step 3: Construct the acceleration search space $A=\{a_{1},a_{2},\ldots ,a_{N_{a}}\}$. For each candidate $a_{k}$, perform BP coherent integration with the exact slant-range model to obtain a sub-image $I_{k}\left(x,y\right)$. Fuse the sub-images by taking the maximum value at each pixel.

Step 4: Apply two-dimensional CFAR detection to the fused spatial image. Estimate the local noise level from the reference cells and compute an adaptive threshold T=τμ, where τ is the CFAR scale factor.

Step 5: Extract local peaks and refine their positions with quadratic surface fitting and a weighted centroid. Output the target list ${\left\{\left({\hat{x}}_{k},{\hat{y}}_{k}\right)\right\}}_{k=1}^{N_{d}}$. For the single-scene comparisons in Section 4, report the detection rate $P_{\det}$ and the conditional localization RMSE over matched detections.

### 3.2. Data-Driven Doppler-to-Spatial Mapping

Backprojection requires a motion model to define the cross-range coordinates. For a candidate pixel with initial cross-range coordinate *x*, the effective cross-range motion is modeled as

(13) $$x\left(t_{m}\right)=x+v_{ref}t_{m}+\frac{1}{2}at_{m}^{2}$$

where *x* is the initial cross-range coordinate, $v_{\mathrm{ref}}$ is the equivalent cross-range reference velocity, and *a* is an effective cross-range acceleration hypothesis. For a candidate reference velocity *v* and acceleration $a_{k}$, the BP response at spatial grid point $\left(x_{i},y_{j}\right)$ is

(14) $$I_{a_{k}}\left(x_{i},y_{j};v\right)=\left|\sum_{m=1}^{M}w_{m}\cdot I_{r}\left\{s;R_{ijkm}\left(v,a_{k}\right),t_{m}\right\}\cdot \exp\left[j\frac{4\pi }{\lambda }R_{ijkm}\left(v,a_{k}\right)\right]\right|$$

where $R_{ijkm}\left(v,a_{k}\right)=\sqrt{{\left({\hat{R}}_{\mathrm{ref}}+y_{j}\right)}^{2}+{\left[x_{i}+vt_{m}+\left(a_{k}/2\right)t_{m}^{2}\right]}^{2}}$ is the exact candidate slant range, $I_{r}\left\{\cdot \right\}$ denotes sampling of the range-compressed data at that generally off-grid range, and $w_{m}$ is the slow-time window.

To compare focusing under different candidate velocities, we define the normalized spatial-energy concentration criterion as

(15) $$J\left(v\right)=\frac{\sum_{i=1}^{N_{x}}\sum_{j=1}^{N_{y}}{\left|I_{\hat{a}\left(i,j\right)}\left(x_{i},y_{j};v\right)\right|}^{4}}{{\left[\sum_{i=1}^{N_{x}}\sum_{j=1}^{N_{y}}{\left|I_{\hat{a}\left(i,j\right)}\left(x_{i},y_{j};v\right)\right|}^{2}\right]}^{2}}$$

where $\hat{a}\left(i,j;v\right)=\arg\max_{a_{k}\in A}\left|I_{a_{k}}\left(x_{i},y_{j};v\right)\right|$ is the locally selected acceleration hypothesis; $I_{\hat{a}\left(i,j;v\right)}$ is therefore the fused response at that grid point. When the candidate velocity matches the equivalent reference velocity, the scattering-center energy is expected to be most concentrated in the spatial domain, so the sharpness measure is maximized.

Multiple scattering centers coexist within a swarm, so interference from nearby targets can create multiple local maxima in the sharpness curve. A coarse velocity prior is therefore added to regularize the sharpness function:

(16) $$J_{\mathrm{reg}}\left(v\right)=\eta_{v}J\left(v\right)+\left(1-\eta_{v}\right)\exp\left[-\frac{{\left(v-v_{0}\right)}^{2}}{2\sigma_{v}^{2}}\right]$$

where $v_{0}$ is the coarse estimate from envelope alignment, $\sigma_{v}$ controls the width of the Gaussian prior, and $\eta_{v}\in \left[0,1\right]$ controls the data–prior balance. Values near one rely mainly on image concentration; smaller values suppress remote local maxima more strongly but make the estimate more sensitive to bias in $v_{0}$.

In this work, the parameters are set to $\eta_{v}=0.65$, $\sigma_{v}=1.0\;m/s$. The coarse estimate $v_{0}$ is obtained from envelope alignment, compared to the true value of 10 m/s. The weight $\eta_{v}=0.65$ ensures that the data-driven sharpness J(v) dominates the objective, while the prior term (weight $1-\eta_{v}=0.35$) effectively suppresses remote local maxima without over-constraining the search. The prior width $\sigma_{v}=1.0\;m/s$ is chosen to match the order of magnitude of the coarse estimation error.

The reference velocity is not a target’s radial translational velocity. Under the far-field approximation, a change in viewing aspect at rate ω gives $v_{\mathrm{ref}}=\omega R_{\mathrm{ref}}$ and $f_{d}≃-2xv_{\mathrm{ref}}/\left(\lambda R_{\mathrm{ref}}\right)$. The data identify the product $\omega R_{\mathrm{ref}}$; ω and $R_{\mathrm{ref}}$ are not separately inferred by the sharpness search. The final estimate maximizes the regularized objective:

(17) $${\hat{v}}_{\mathrm{ref}}=\arg\max_{v\in V}J_{\mathrm{reg}}\left(v\right)$$

where V is the candidate-velocity grid. Once ${\hat{v}}_{\mathrm{ref}}$ is estimated, the mapping coefficient is $\kappa =\lambda {\hat{R}}_{\mathrm{ref}}/\left(2{\hat{v}}_{\mathrm{ref}}\right)$ and $\hat{x}=-\kappa f_{d}$. Its first-order relative sensitivity is

(18) $$\frac{\delta \kappa }{\kappa }≃\frac{\delta R_{\mathrm{ref}}}{R_{\mathrm{ref}}}-\frac{\delta v_{\mathrm{ref}}}{v_{\mathrm{ref}}}$$

Consequently, stable mapping requires a resolvable change in viewing aspect over the coherent interval. When $|v_{\mathrm{ref}}|$ is small, this condition can be improved through a longer aperture or an additional angular constraint. Supplementary Figure S1a,b and Supplementary Table S1 quantify the effects of the true equivalent reference velocity and an imposed reference-velocity mismatch, respectively.

Figure 4a shows a 51-point candidate sweep from 7.5 to 12.5 m/s in 0.1 m/s steps. The regularized criterion reaches its sampled maximum at approximately 9.10 m/s, close to the simulation reference of 10 m/s. Figure 4b shows the resulting Doppler-to-cross-range calibration and recovered spatial distribution, illustrating how the estimated reference velocity enters the coordinate mapping.

### 3.3. BP with Acceleration Grid Search

BP back-projects the radar echoes along candidate slant-range histories. Coherent integration across all pulses then forms a focused image. Here, *a* denotes the target-dependent effective cross-range acceleration in Equation (1), rather than bulk radial acceleration or the scene-level acceleration scale used in Section 4. For a spatial grid point (x,y), the candidate slant range at slow time $t_{m}$ is

(19) $$R\left(x,y;t_{m};a\right)=\sqrt{{\left({\hat{R}}_{ref}+y\right)}^{2}+{\left(x+{\hat{v}}_{ref}t_{m}+\frac{1}{2}at_{m}^{2}\right)}^{2}}$$

where ${\hat{v}}_{\mathrm{ref}}$ is estimated as described in Section 3.2, and *a* is a candidate acceleration. Under hypothesis $a_{k}$, coherent integration is written as

(20) $$I_{a_{k}}\left(x,y\right)=\left|\sum_{m=1}^{M}w_{m}\;I_{r}\left\{s;R\left(x,y;t_{m};a_{k}\right),t_{m}\right\}\exp\left[j\frac{4\pi }{\lambda }R\left(x,y;t_{m};a_{k}\right)\right]\right|$$

Here, $w_{m}$ is the slow-time window. The complete phase factor $\exp[j4\pi R\left(x,y;t_{m};a_{k}\right)/\lambda ]$ compensates the candidate range-dependent phase so that matched pulse contributions add coherently. The generally off-grid range from Equation (19) is evaluated by the interpolation operator introduced in Equation (14).

To represent the different accelerations of sub-targets, we construct the following search grid:

(21) $$A=\left\{a_{\min}+\left(k-1\right)\Delta a:\;k=1,\ldots ,N_{a}\right\},\;\;N_{a}=1+\left\lfloor \frac{a_{\max}-a_{\min}}{\Delta a}\right\rfloor$$

For each candidate acceleration $a_{k}$, Equation (20) produces a sub-image $I_{a_{k}}\left(x,y\right)$. A target forms a focused peak when the candidate matches its effective acceleration. A mismatch spreads that target’s energy into the background. The $N_{a}$ sub-images are fused by taking the maximum at each pixel:

(22) $$I\left(x,y\right)=\max_{k=1,\ldots ,N_{a}}\;I_{a_{k}}\left(x,y\right)$$

The pixel-wise maximum selects the locally best-matched acceleration hypothesis and thereby compensates target-dependent motion without assigning a single acceleration to the whole swarm. Maximization also raises noise peaks. If the $N_{a}$ noise-only sub-images were independent and a single sub-image threshold had exceedance probability $P_{\mathrm{FA},1}$, then the fused-image exceedance probability would be $1-{\left(1-P_{\mathrm{FA},1}\right)}^{N_{a}}$. The actual sub-images are correlated, so the fused noise distribution or the final CFAR threshold should be calibrated empirically rather than copied from one sub-image. Sum fusion may blur mismatched responses, whereas maximum fusion preserves a matched local peak. The controlled ablation in Section 4.4 quantifies this trade-off at independently calibrated noise-only operating points.

For $N_{v}$ trial reference velocities, $N_{a}$ acceleration hypotheses, *M* pulses, and an $N_{x}\times N_{y}$ spatial grid, direct evaluation has complexity $O(N_{v}N_{a}MN_{x}N_{y})$. Once ${\hat{v}}_{\mathrm{ref}}$ is fixed, image formation costs $O(N_{a}MN_{x}N_{y})$. Streaming the running maximum avoids storing all sub-images and needs $O(N_{x}N_{y})$ image memory. Coarse-to-fine motion grids, GPU parallelization over pixels and hypotheses, and early pruning of low-response hypotheses are practical acceleration strategies. Section 4.4 reports component-level timing across the tested acceleration-grid densities.

BP matches the predicted range-dependent phase directly rather than assigning position from a single folded Doppler bin. Supplementary Figure S1c,d shows that the nominal 1 kHz configuration lies within the unaliased region and illustrates the transition under the separate high-velocity stress condition.

### 3.4. CFAR Detection and Localization

The fused BP image is searched for focused target responses, which are then localized by peak refinement.

Two-dimensional CFAR detection is applied to the fused image. A reference window around each cell under test contains guard and reference cells; guard cells reduce contamination of the noise estimate by target energy. For CA-CFAR, the local noise-level estimate is

(23) $$\mu =\frac{1}{N_{\mathrm{ref}}}\sum_{\left(p,q\right)\in \Omega_{\mathrm{ref}}}I_{p,q}$$

where $\Omega_{\mathrm{ref}}$ is the set of reference cells and $N_{\mathrm{ref}}$ is its cardinality. The adaptive threshold is

(24) T=τμ

where τ is the scale factor for the desired false-alarm behavior after maximum fusion. A cell becomes a candidate when $I_{i,j}\geq T$.

Local maxima are extracted from the cells above the threshold. A sliding window keeps only the strongest point in each neighborhood. This step removes adjacent secondary peaks and produces the candidate target positions.

To improve localization, a weighted centroid is calculated around each candidate. For a peak at $\left(x_{k},y_{k}\right)$, the energy centroid within a local window $\Omega_{k}$ is

(25) $${\hat{x}}_{\mathrm{centroid}}\;=\frac{\sum_{\left(x,y\right)\in \Omega_{k}}x\cdot I^{2}\left(x,y\right)}{\sum_{\left(x,y\right)\in \Omega_{k}}I^{2}\left(x,y\right)},\;{\hat{y}}_{\mathrm{centroid}}\;=\frac{\sum_{\left(x,y\right)\in \Omega_{k}}y\cdot I^{2}\left(x,y\right)}{\sum_{\left(x,y\right)\in \Omega_{k}}I^{2}\left(x,y\right)}$$

where $\Omega_{k}$ is the local neighborhood around the peak. Enumerate the $L=|\Omega_{k}|$ samples in the fitting neighborhood as $\left(x_{\ell },y_{\ell }\right)$, ℓ=1,…,L, and define

(26) $$z_{\ell }=\ln\left[I\left(x_{\ell },y_{\ell }\right)+\epsilon \right],\;\;\ell =1,\ldots ,L$$

where ϵ is a small positive constant that prevents a logarithmic singularity. The quadratic surface is

(27) $$z\left(x,y\right)=a_{0}+a_{1}x+a_{2}y+a_{3}x^{2}+a_{4}xy+a_{5}y^{2}$$

In matrix form:

(28) z=Ha

where

(29) $$z=\left[\begin{array}{c} z_{1}\\ z_{2}\\ \vdots \\ z_{L} \end{array}\right],\;a=\left[\begin{array}{c} a_{0}\\ a_{1}\\ a_{2}\\ a_{3}\\ a_{4}\\ a_{5} \end{array}\right]$$

(30) $$H=\left[\begin{array}{cccccc} 1 & x_{1} & y_{1} & x_{1}^{2} & x_{1}y_{1} & y_{1}^{2}\\ 1 & x_{2} & y_{2} & x_{2}^{2} & x_{2}y_{2} & y_{2}^{2}\\ \vdots & \vdots & \vdots & \vdots & \vdots & \vdots \\ 1 & x_{L} & y_{L} & x_{L}^{2} & x_{L}y_{L} & y_{L}^{2} \end{array}\right]$$

The grid points in the neighborhood are fitted by least squares:

(31) $$\hat{a}=\arg\min_{a}\;{∥z-\mathrm{Ha}∥}_{2}^{2}$$

The closed-form solution is

(32) $$\hat{a}={\left(H^{T}H\right)}^{-1}H^{T}z$$

The extremum of the fitted surface gives the refined position. We differentiate Equation (27) with respect to *x* and *y* and set both derivatives to zero:

(33) $$\frac{\partial z}{\partial x}=a_{1}+2a_{3}x+a_{4}y=0,\;\frac{\partial z}{\partial y}=a_{2}+a_{4}x+2a_{5}y=0$$

Solving these equations gives the extremum position:

(34) $$\left[\begin{array}{cc} 2a_{3} & a_{4}\\ a_{4} & 2a_{5} \end{array}\right]\left[\begin{array}{c} {\hat{x}}_{\mathrm{quad}}\\ {\hat{y}}_{\mathrm{quad}} \end{array}\right]=-\left[\begin{array}{c} a_{1}\\ a_{2} \end{array}\right]$$

Let $Q=\left[\begin{array}{cc} 2a_{3} & a_{4}\\ a_{4} & 2a_{5} \end{array}\right]$ be the Hessian of the fitted log-amplitude surface. The quadratic extremum is accepted only when Q is negative definite and the solution lies inside the fitting neighborhood; otherwise, the centroid is used as a stable fallback. The final position is a weighted combination of the two valid estimates:

(35) $$\left[\begin{array}{c} \hat{x}\\ \hat{y} \end{array}\right]=\eta_{p}\left[\begin{array}{c} {\hat{x}}_{\mathrm{quad}}\\ {\hat{y}}_{\mathrm{quad}} \end{array}\right]+\left(1-\eta_{p}\right)\left[\begin{array}{c} {\hat{x}}_{\mathrm{centroid}}\\ {\hat{y}}_{\mathrm{centroid}\;} \end{array}\right]$$

where $\eta_{p}\in \left[0,1\right]$ controls the contribution of each estimator.

For the fixed-formation experiments in Section 4, let $N_{\mathrm{match}}$ be the number of one-to-one matched detections among $N_{t}=15$ true targets. The scene-level detection rate is

(36) $$P_{\det}=\frac{N_{\mathrm{match}}}{N_{t}}$$

The reported localization error is conditional on matched detections:

(37) $${\mathrm{RMSE}}_{\mathrm{match}}=\sqrt{\frac{1}{N_{\mathrm{match}}}\sum_{\ell =1}^{N_{\mathrm{match}}}\left[{\left({\hat{x}}_{\ell }-x_{\ell }\right)}^{2}+{\left({\hat{y}}_{\ell }-y_{\ell }\right)}^{2}\right]}$$

The fixed-formation values are integer multiples of 1/15, so $P_{\det}$ is reported as a single-scene detection rate rather than a Monte Carlo probability. Section 4.4 separately reports repeated-scene probabilities and scene-bootstrap confidence intervals under the stated association and threshold rules.

## 4. Simulation Results and Analysis

### 4.1. Radar Parameter Configuration

The radar and fixed-formation settings are summarized in Table 1.

The simulation uses the irregular UAV formation shown in Figure 5a. Figure 5b projects the formation onto the two-dimensional radar plane (z=0). The formation contains $N_{t}=15$ point targets in a single fixed cluster and spans approximately −50 to 50 m in both cross-range and range. The common equivalent cross-range reference velocity is $v_{\mathrm{ref}}=10$ m/s. Individual effective cross-range accelerations are generated as

(38) $$a_{i}=a_{s}\sin\left(0.71i\right)+\varepsilon_{i},\;\;i=1,\ldots ,15$$

where $\varepsilon_{i}$ is a target-dependent perturbation, and $a_{s}$ is a scale parameter rather than the arithmetic mean of the signed accelerations. In the $a_{s}=5.4$ m/s^2^ scene shown in Figure 5b, the target accelerations span approximately −5.22 to 5.39 m/s^2^.

Figure 6a shows the range-compressed echo map. The envelopes curve in the range–slow-time plane, and energy from one scattering center spreads across several range cells. Echoes in the dense central region also lie close together. After envelope alignment, each target trace in Figure 6b becomes approximately stationary in its own range cell.

### 4.2. Resolution Results

Figure 7 compares the resolution images at $a_{s}=1.8$, 5.4, 7.2, and 9 m/s^2^. Each row represents one acceleration scale. From left to right, the columns show RD, second-order Taylor-Chirplet, third-order Taylor-Chirplet, and the proposed method. White circles mark the true target projections, and colored peaks show the focused responses. In this scene, RD produces broader, more dispersed responses, and several nearby targets overlap. The Taylor-Chirplet variants improve focusing but retain unresolved or missed targets at the larger acceleration scales. The proposed method produces more compact responses and preserves more of the closely spaced targets. Section 4.4 reports the repeated-scene SNR and Swerling-I results.

### 4.3. Localization Scatter Plots

At $a_{s}=7.2$ m/s^2^, RD, second-order Taylor-Chirplet, third-order Taylor-Chirplet, and the proposed method detect 11, 10, 10, and 13 of the 15 targets, respectively (Figure 8). Their conditional two-dimensional localization RMSE values are 3.36, 2.63, 2.30, and 1.12 m, and no false alarms are observed in this scene. Relative to RD, the proposed method reduces the conditional RMSE by approximately 67% while detecting two additional targets.

Table 2 summarizes the five single-scene results as $a_{s}$ increases from 1.8 to 9 m/s^2^. The proposed method has the lowest conditional two-dimensional RMSE at every tested scale. At $a_{s}=7.2$ and 9 m/s^2^, it yields 13 matched detections, whereas the baseline methods yield 8–11. Because missed targets are excluded from RMSE, this measure is interpreted together with $P_{\det}$.

### 4.4. Monte Carlo Sensitivity Analysis

To assess performance beyond the fixed formation, we conducted Monte Carlo simulations using the exact slant-range model in Equations (19)–(22). The experiments examine acceleration-grid spacing, fusion rule, SNR, Swerling-I scattering, PRF, and reference-velocity mismatch. Except for the controlled variables, the nominal radar settings follow Table 1. The simulations use a Hann slow-time window, unit-power circular complex Gaussian noise, and one Swerling-I power draw per target and CPI. Each trial contains 15 targets, with noise, target amplitude, and small position and acceleration perturbations resampled between trials; paired draws are used when processing rules are compared. Range-gated local windows isolate the motion-compensation and fusion mechanisms from range-cell interactions.

For computationally matched sensitivity sweeps, 256 indices are selected uniformly from the 1024-pulse record, including its first and last samples; the selected subset therefore spans 1.023 s of the 1.024 s record. The reported timing measures this 256-sample local-window component and is not an end-to-end time for full-image processing with all 1024 pulses.

A matched detection requires both an empirical noise-only threshold exceedance and a cross-range error not exceeding 3 m. Conditional cross-range RMSE, denoted ${\mathrm{RMSE}}_{x}$, is calculated over matched detections and is distinct from the two-dimensional fixed-scene metric in Equation (37). The miss-penalized RMS assigns 5 m to each missed or unassociated target. The 95% performance intervals use 2000 complete-scene bootstrap resamples, preserving within-scene dependence; Wilson intervals are used for the noise-only exceedance probabilities. Table 3 summarizes the two main-text experiments, while the PRF and reference-velocity study is reported in Supplementary Figure S1 and Table S1.

#### 4.4.1. Acceleration-Grid and Fusion Sensitivity

The acceleration-grid experiment compares pixel-wise maximum fusion, mean fusion, and a zero-acceleration hypothesis over the settings in Table 3. For every grid and processing rule, the empirical threshold is calibrated from noise-only windows and evaluated on a disjoint noise-only split at a target gate-level exceedance probability of 0.01.

As shown in Figure 9, at Δa=4 m/s^2^, maximum fusion achieves a matched-detection probability of 0.499 (95% CI: 0.472–0.527), compared with 0.311 (0.293–0.330) for mean fusion and 0.270 (0.253–0.288) for the zero-acceleration hypothesis. The corresponding ${\mathrm{RMSE}}_{x}$ values are 1.631, 1.700, and 1.618 m, respectively. The gain is therefore primarily in matched detection, with similar conditional cross-range error.

Grid refinement below Δa=4 m/s^2^ does not improve matched detection in this setting. For maximum fusion, the matched-detection probability decreases from 0.499 at Δa=4 m/s^2^ to 0.420 at 0.25 m/s^2^, while the local-window time increases from 6.6 to 94.4 ms. The independently validated noise-only exceedance probability remains close to the 0.01 target (0.0113–0.0125). Thus, refining the grid below Δa=4 m/s^2^ does not improve matched detection in this experiment and increases the measured component time.

#### 4.4.2. SNR and Swerling-I Sensitivity

The SNR experiment fixes Δa=4 m/s^2^ and evaluates mean per-pulse input SNRs of −18, −15, −12, −9, and −6 dB. For each SNR, 200 paired scenes provide 3000 target windows. One Swerling-I power sample is drawn for each target and held constant within its CPI. The threshold for each processing rule is calibrated once from 6000 noise-only windows and then held fixed over the complete SNR sweep; another 6000 windows are used for independent validation.

Figure 10 shows that maximum fusion provides the highest matched-detection probability and the lowest miss-penalized RMS error at every tested SNR. From −18 to −6 dB, its matched-detection probability increases from 0.124 to 0.598, compared with 0.097 to 0.411 for mean fusion and 0.077 to 0.329 for the zero-acceleration hypothesis. At −9 dB, the respective probabilities are 0.503 (95% CI: 0.487–0.519), 0.356 (0.341–0.371), and 0.274 (0.262–0.286); the corresponding miss-penalized RMS errors are 3.706, 4.140, and 4.344 m.

The ${\mathrm{RMSE}}_{x}$ of maximum fusion remains between 1.556 and 1.717 m over the SNR sweep while the number of matched targets increases. Because conditional RMSE excludes missed targets, it is interpreted jointly with matched-detection probability and the miss-penalized RMS. The independently validated noise-only exceedance rates are 0.0095, 0.0102, and 0.0097 for maximum fusion, mean fusion, and the zero-acceleration hypothesis. Their similarity confirms that the detection comparison is made at comparable noise-only thresholds.

#### 4.4.3. PRF and Reference-Velocity Operating Region

Supplementary Figure S1 reports the true-reference-velocity and PRF sweeps, and Supplementary Table S1 reports imposed reference-velocity mismatch. At the nominal setting of $v_{\mathrm{ref}}=10$ m/s and PRF =1 kHz, the normalized Doppler occupancy $2f_{\max}/\mathrm{PRF}$ is 0.0107 and the maximum range migration is approximately 0.071 range cells. The nominal configuration is therefore well within the unaliased sampling region. When the true velocity is 10 m/s, imposed reference-velocity errors of −5%, 0, and +5% give matched-detection probabilities of 0.588, 0.696, and 0.651, respectively; errors of −10% and +10% give 0.392 and 0.489. A separate high-velocity condition in Supplementary Figure S1 shows the transition between folded and unaliased PRF regimes.

## 5. Discussion and Scope

The fixed-formation experiments compare localization performance with RD and Taylor-Chirplet baselines. The Monte Carlo experiments evaluate fusion, grid spacing, SNR, PRF, and reference-velocity sensitivity under repeated noise and Swerling-I realizations. The validated noise-only exceedance rates remain close to their calibration targets, and scene-level bootstrap intervals quantify variation across trials.

The operating-region results show reduced cross-range observability at small $|v_{\mathrm{ref}}|$, while the nominal PRF retains a wide unaliased sampling margin. The measured local-window times describe relative scaling with acceleration-grid density; full-image scaling is given by the complexity analysis in Section 3. These conclusions apply to the tested point-target model, formation geometries, and radar settings. Future work may introduce more realistic scattering models and micro-Doppler features to further assess performance in complex electromagnetic environments.

## 6. Conclusions

This paper proposed a lateral-Doppler localization method that estimates the equivalent cross-range reference velocity from spatial concentration and compensates target-dependent motion through acceleration-hypothesis BP and pixel-wise maximum fusion. The echo data determine the rotation-related product $v_{\mathrm{ref}}=\omega R_{\mathrm{ref}}$ rather than requiring an externally supplied rotation rate.

In the fixed-formation experiments, the method produced more compact responses, more matched detections, and lower conditional two-dimensional localization RMSE than the RD and Taylor-Chirplet baselines, particularly at the larger acceleration scales. In the Monte Carlo experiments, maximum fusion yielded the highest matched-detection probability and lowest miss-penalized RMS error among the tested fusion strategies across per-pulse SNRs from −18 to −6 dB at comparable noise-only thresholds. Refining the tested acceleration grid below Δa=4 m/s^2^ did not improve matched detection and increased the measured component time. The PRF and reference-velocity sweeps also identified the nominal unaliased operating point and quantified sensitivity to velocity mismatch.

## Figures and Tables

**Figure 1:**
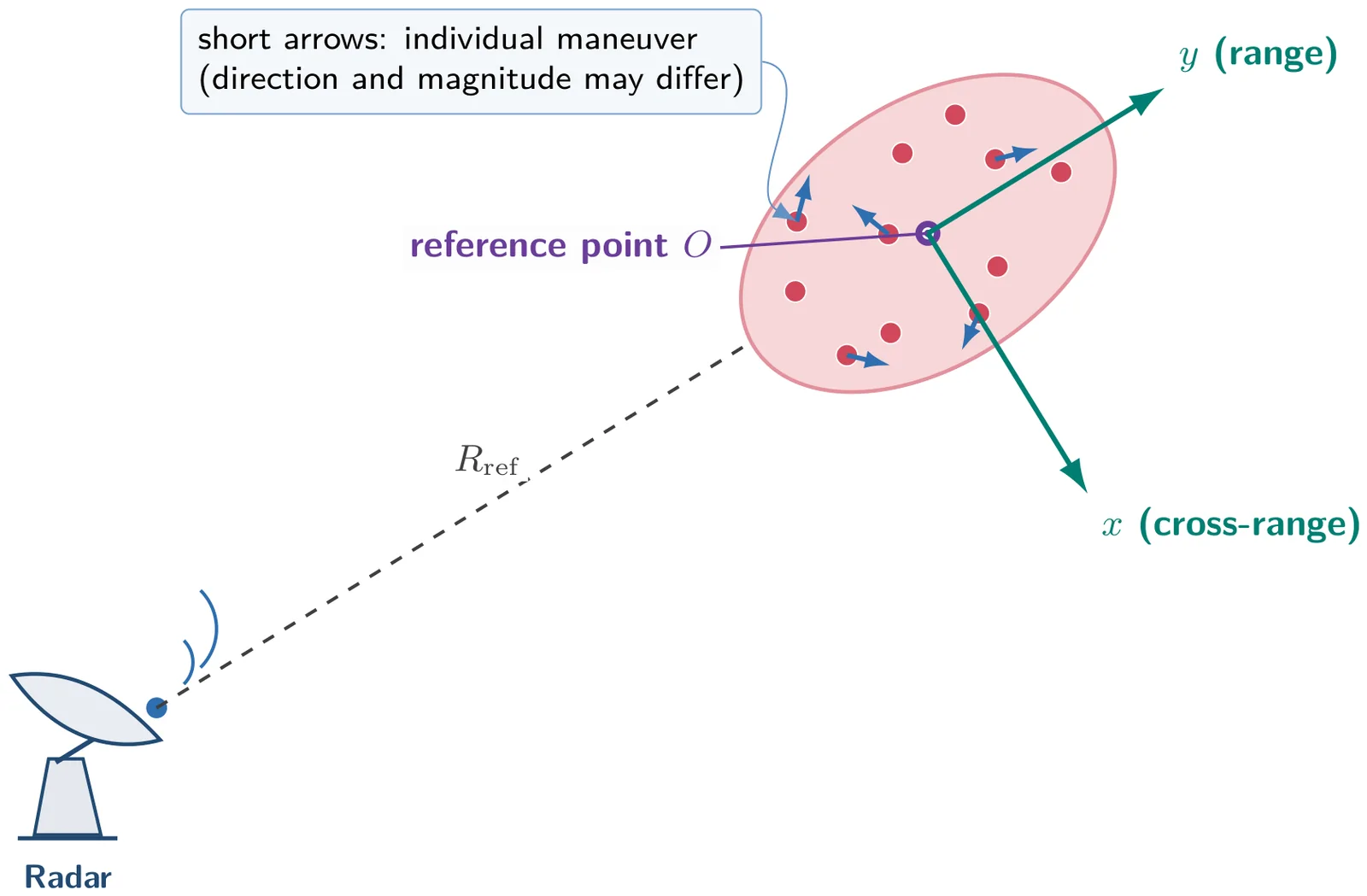
Observation geometry and local coordinates for a UAV swarm. The *y*-axis is aligned with the radar line of sight and the *x*-axis is the cross-range direction. The short blue arrows denote the individual maneuver vectors of sub-targets in the swarm.

**Figure 2:**
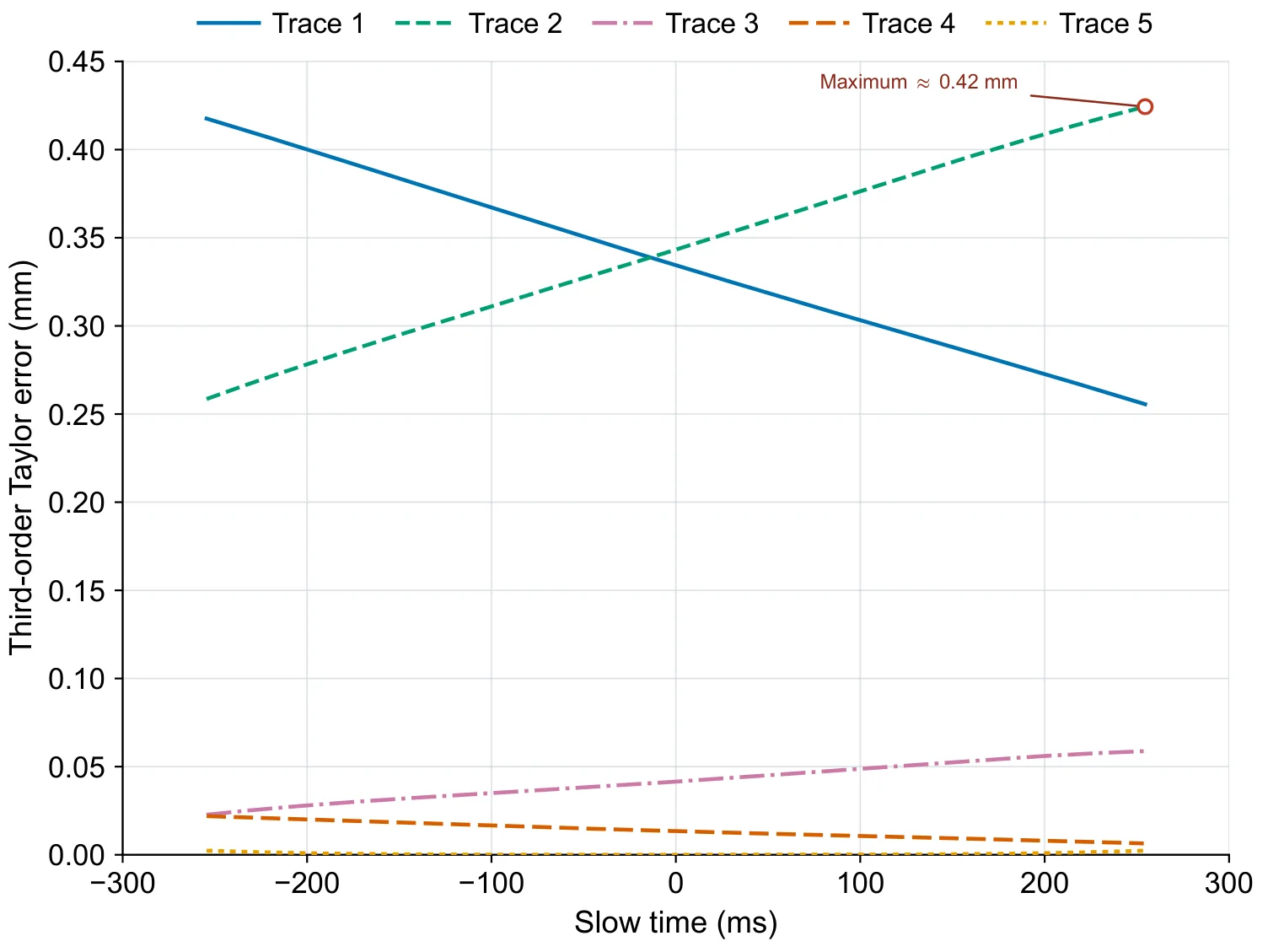
Absolute slant-range error of the third-order Taylor approximation over the displayed slow-time interval.

**Figure 3:**
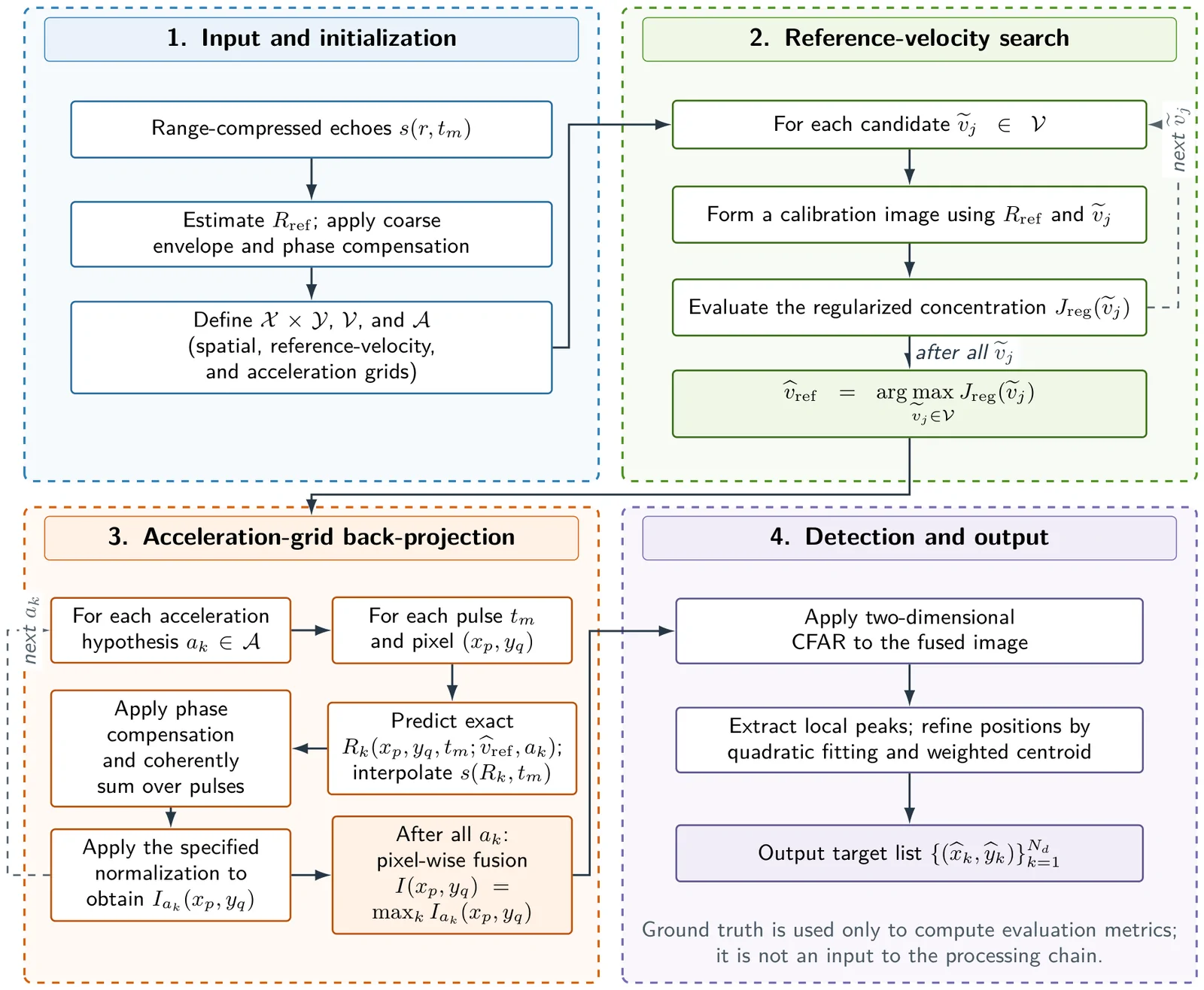
Processing flow of the proposed lateral-Doppler localization method, including reference-velocity search, acceleration-grid BP, pixel-wise fusion, detection, and position refinement.

**Figure 4:**
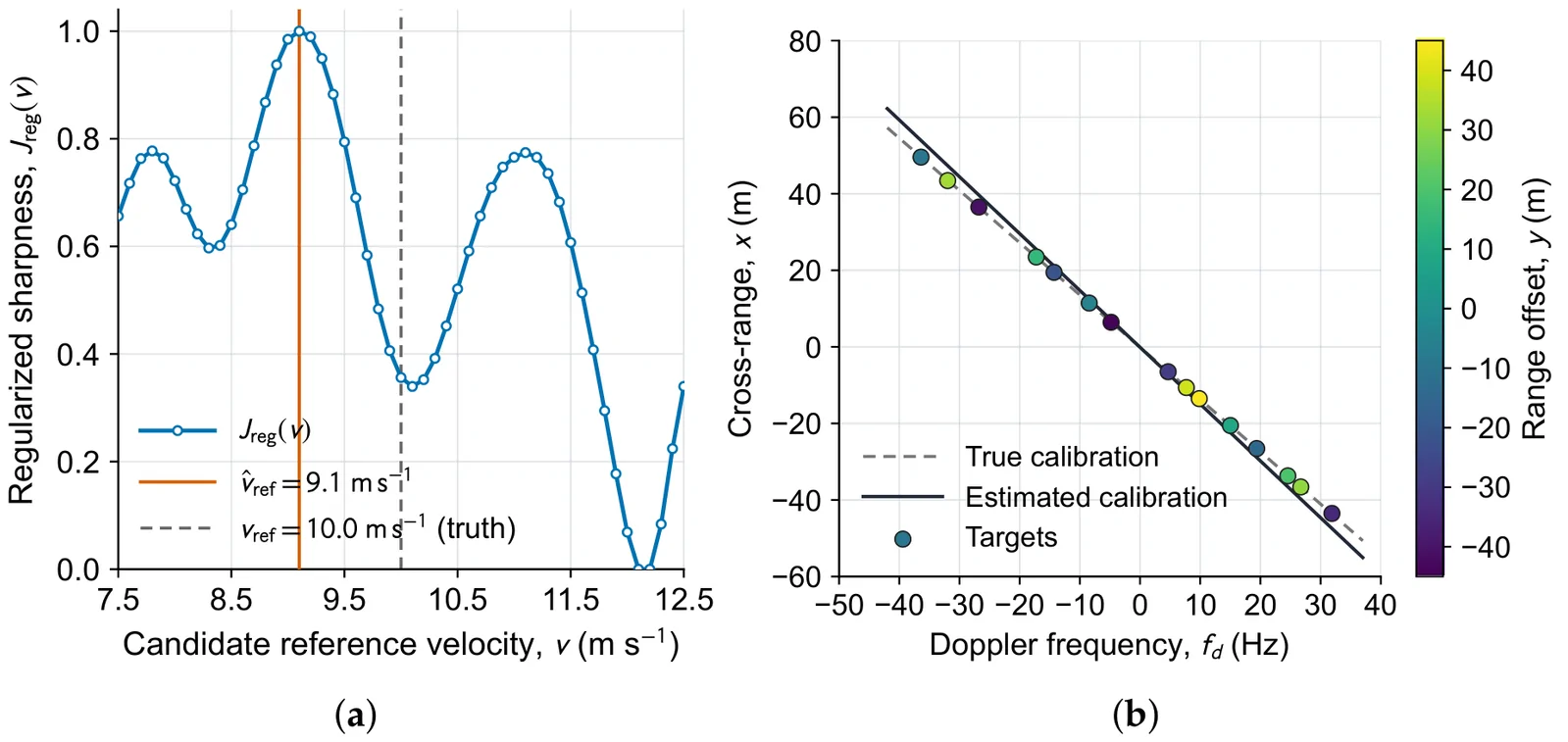
(**a**) Regularized spatial-concentration criterion versus candidate reference velocity. (**b**) Doppler-to-cross-range calibration and recovered target distribution.

**Figure 5:**
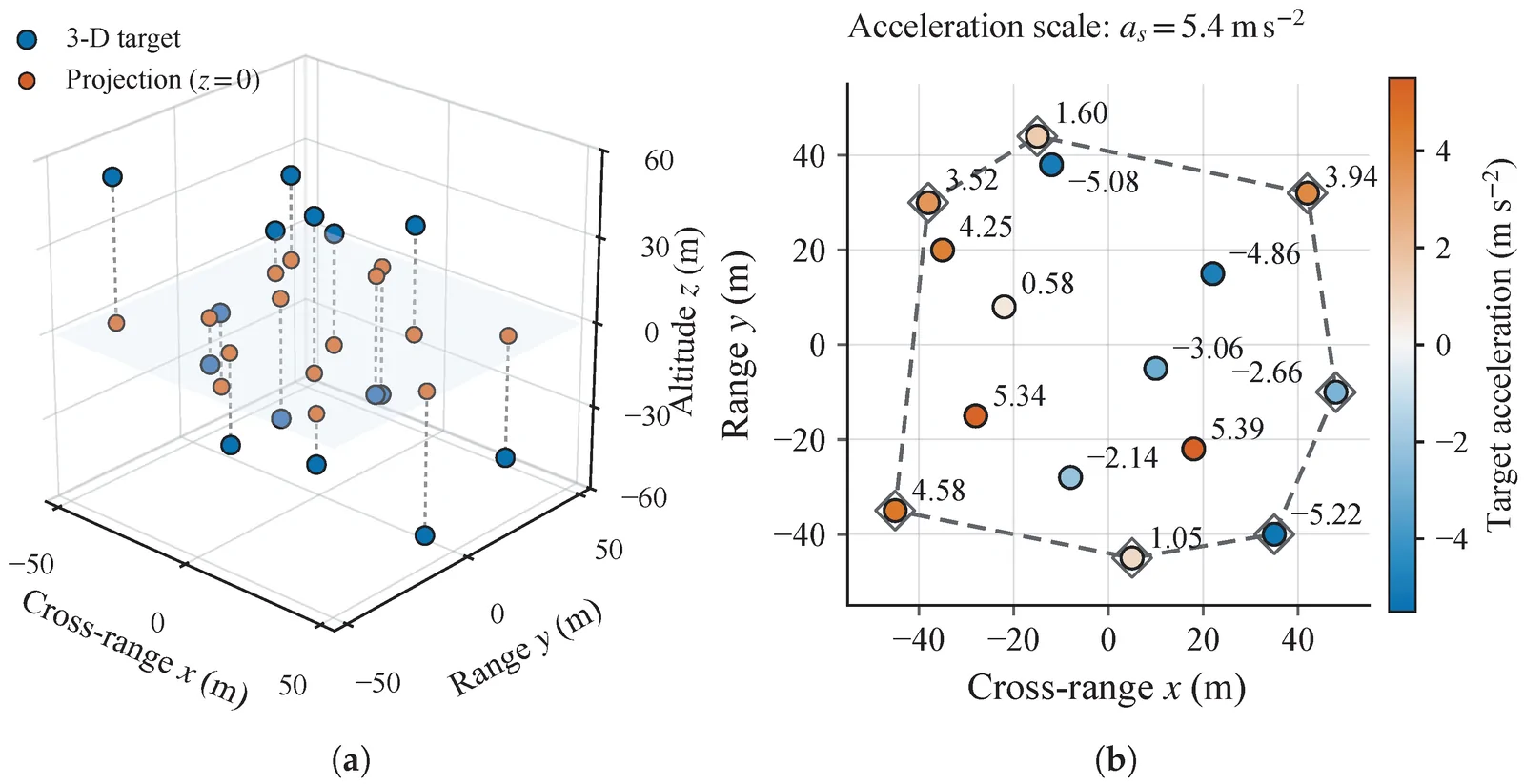
(**a**) Irregular formation model. (**b**) Two-dimensional projection. The dashed line represents the outer boundary of the swarm formation, and the gray boxes highlight the outermost targets.

**Figure 6:**
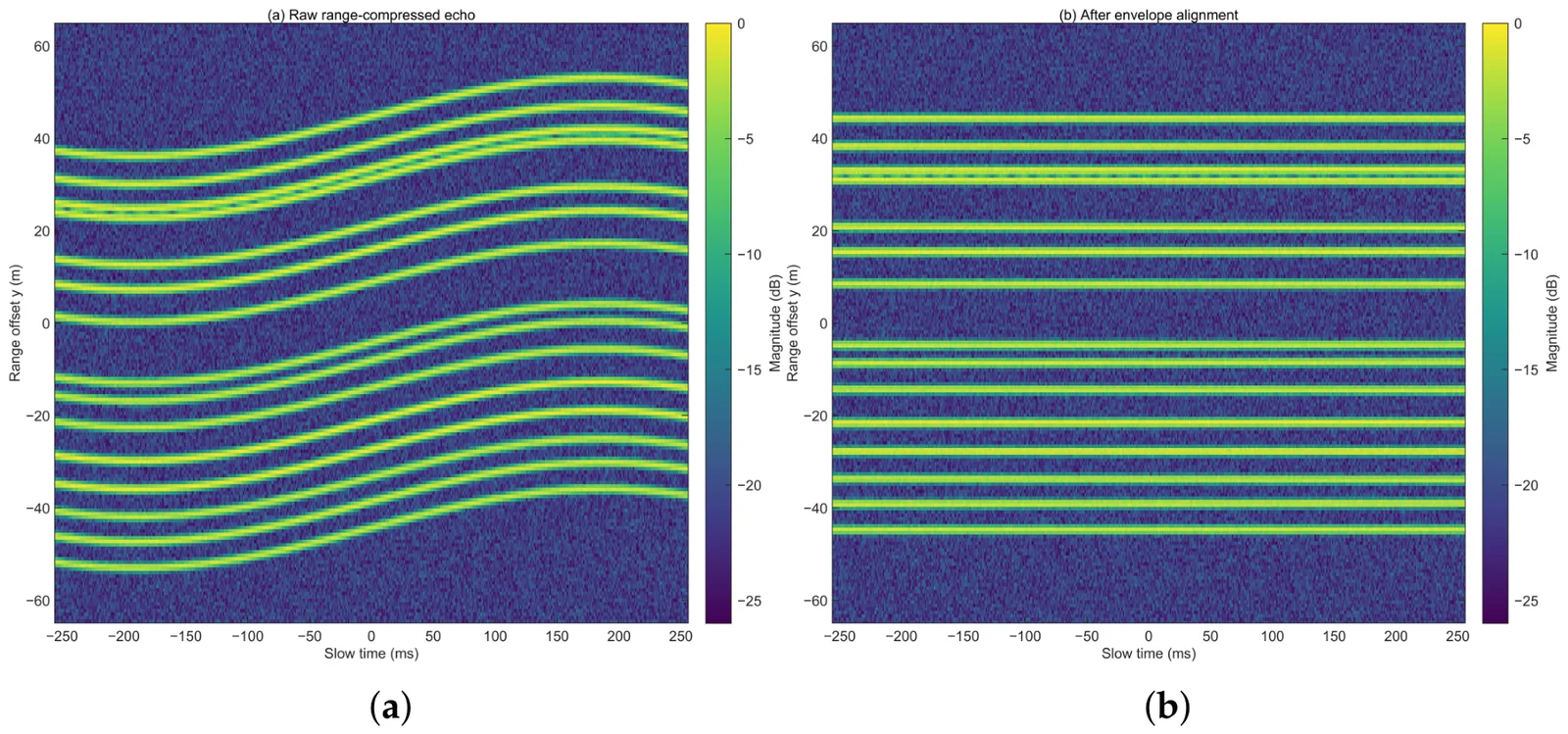
(**a**) Echoes after range compression. (**b**) Echoes after envelope alignment. The color represents the echo amplitude.

**Figure 7:**
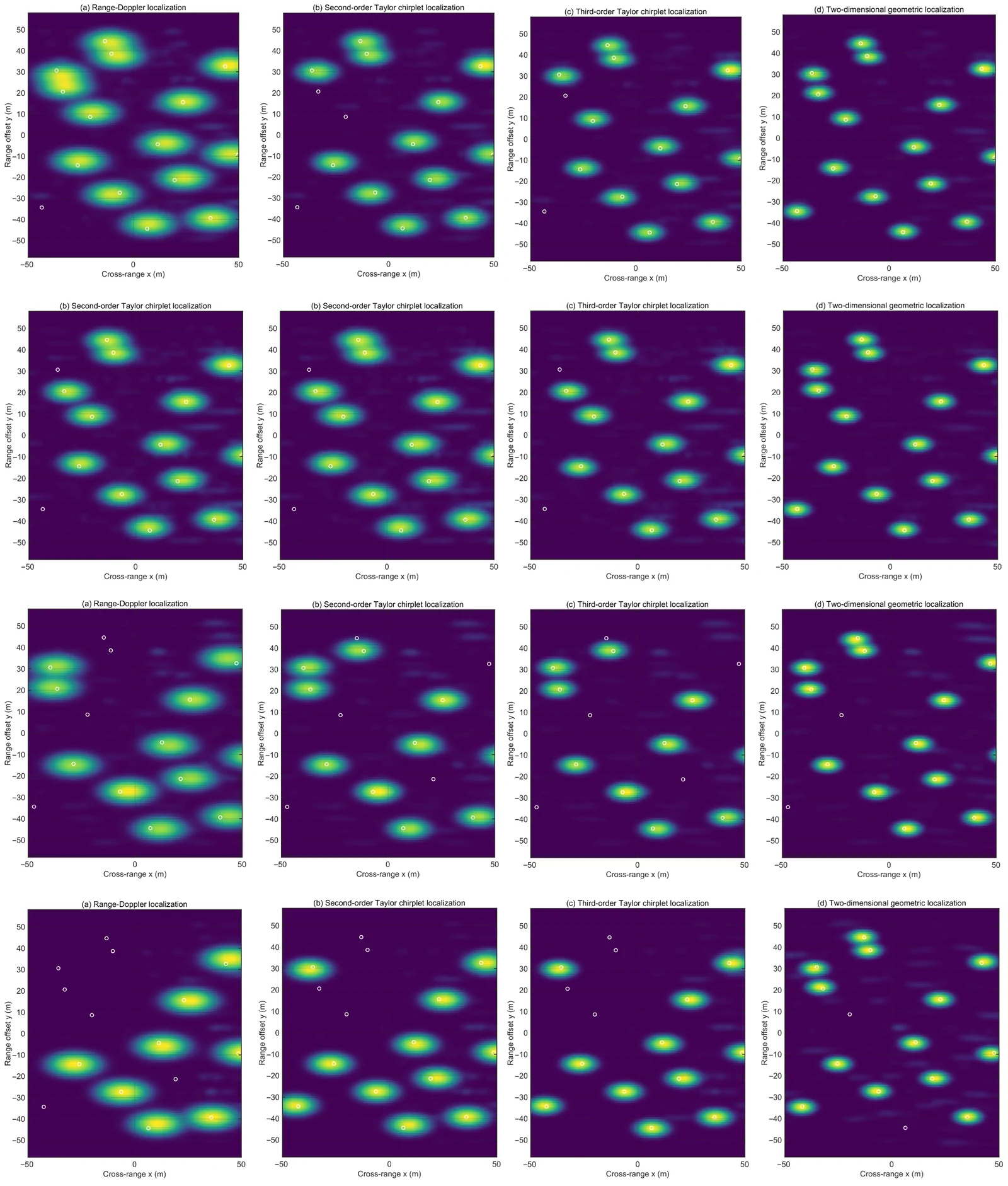
Resolution results at acceleration scales $a_{s}=1.8$, 5.4, 7.2, and 9 m/s^2^. The color represents the normalized echo amplitude.

**Figure 8:**
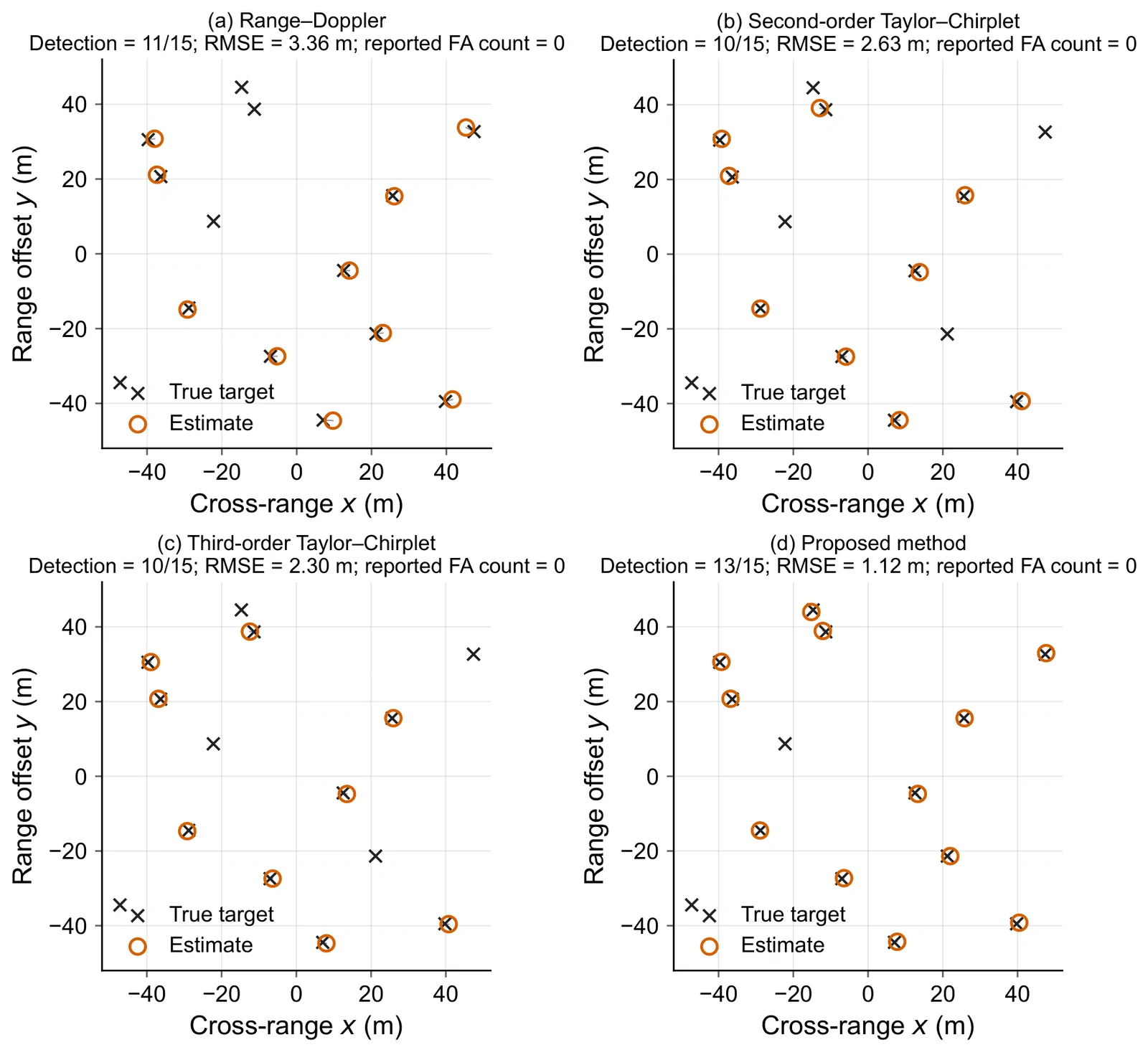
Single-scene localization results of RD, second-order Taylor-Chirplet, third-order Taylor-Chirplet, and the proposed method at acceleration scale $a_{s}=7.2$ m/s^2^.

**Figure 9:**
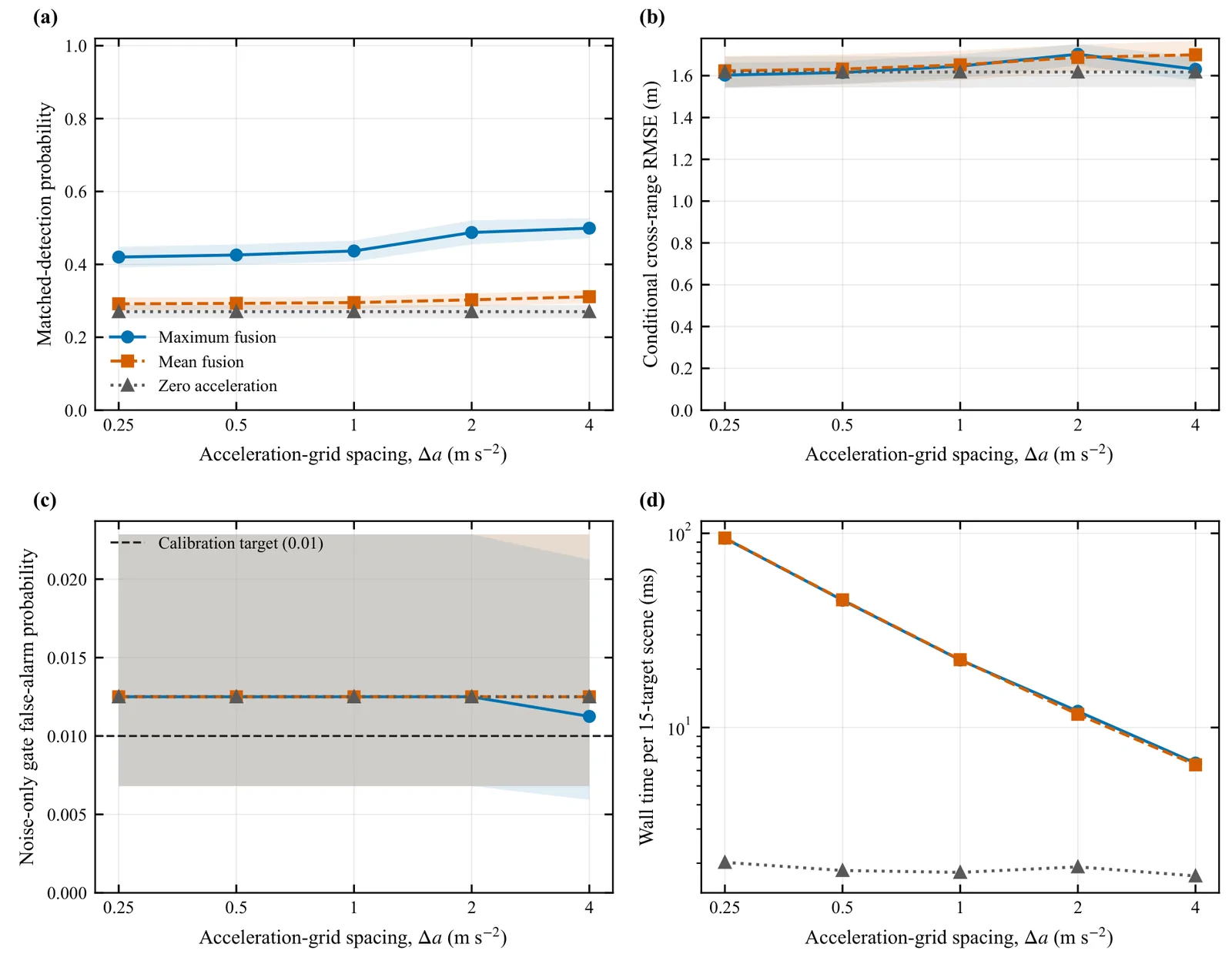
Acceleration-grid and fusion sensitivity. (**a**) Matched-detection probability; (**b**) conditional cross-range RMSE (${\mathrm{RMSE}}_{x}$) over matched detections; (**c**) noise-only gate exceedance probability on an independent validation split (dashed line: 0.01 calibration target); and (**d**) component wall time per 15-target scene using 256 slow-time samples. Shading shows 95% scene-bootstrap intervals in (**a**,**b**) and Wilson intervals in (**c**).

**Figure 10:**
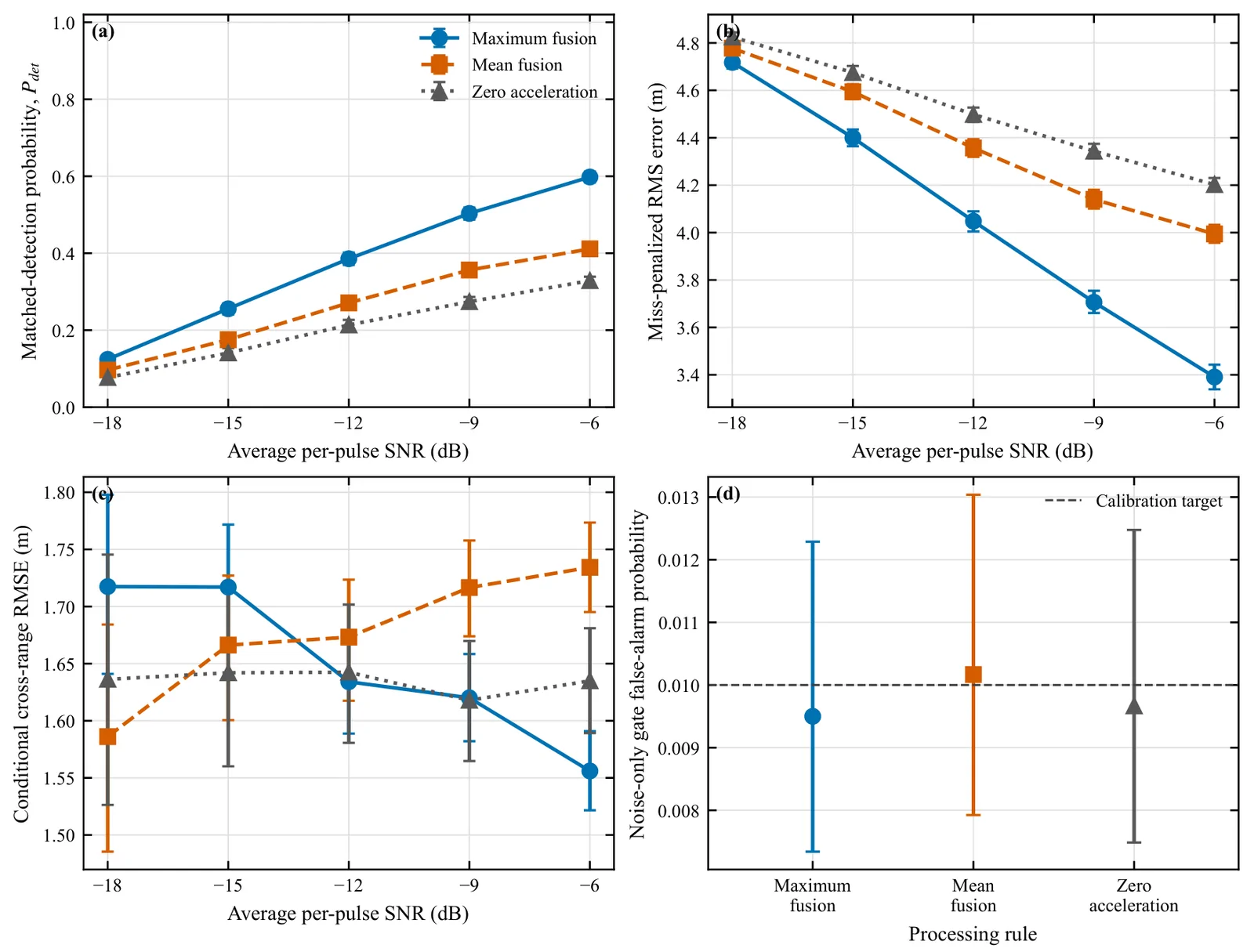
SNR sensitivity under Swerling-I scattering. (**a**) Matched-detection probability; (**b**) miss-penalized RMS error with a 5 m penalty; (**c**) conditional cross-range RMSE (${\mathrm{RMSE}}_{x}$) over matched detections; and (**d**) noise-only gate exceedance probability on an independent validation split. Thresholds are fixed across SNR. Error bars show 95% scene-bootstrap intervals in (**a**–**c**) and Wilson intervals in (**d**).

**Table 1:** Radar and fixed-formation simulation parameters.

Parameter	Value
Carrier frequency	11 GHz
Signal bandwidth	200 MHz
Pulse repetition frequency	1 kHz
Number of coherently integrated pulses	1024
Detection range	10 km
Integration time	1.024 s
Range resolution	0.75 m
Number of point targets	15
Equivalent cross-range reference velocity	10 m/s
Approximate formation extent	100 m×100 m
Acceleration scales $a_{s}$	1.8, 3.6, 5.4, 7.2, and 9 m/s^2^

**Table 2:** Single-scene detection rate and conditional two-dimensional localization RMSE versus acceleration scale.

	RD		Second-Order Taylor-Chirplet		Third-Order Taylor-Chirplet		Proposed Method	
$a_{s}$ (m/s^2^)	$P_{\det}$	RMSE (m)	$P_{\det}$	RMSE (m)	$P_{\det}$	RMSE (m)	$P_{\det}$	RMSE (m)
1.8	1	2.61	1	1.51	1	1.05	**1**	**0.81**
3.6	0.93	2.84	0.8	1.88	0.87	1.4	**1**	**0.95**
5.4	0.87	3.23	0.87	2.09	0.87	1.54	**1**	**0.96**
7.2	0.73	3.36	0.67	2.63	0.67	2.3	**0.87**	**1.12**
9	0.53	3.98	0.73	3.71	0.73	2.82	**0.87**	**1.15**

$P_{\det}=N_{\mathrm{match}}/15$ for one simulated formation. RMSE is the two-dimensional position error in Equation (37), calculated only over one-to-one matched detections; it does not penalize missed targets. Values in bold denote results of the proposed method.

**Table 3:** Design of the grid/fusion and SNR Monte Carlo experiments.

Experiment	Slow-Time Record	Controlled Variation	Scenes; Noise Windows
Grid/fusion	256-sample subset spanning 1.023 s	SNR =−9.5 dB; a∈[−10,10] m/s^2^; Δa∈{4,2,1,0.5,0.25} m/s^2^	96 scenes; 800/800 per grid and rule
SNR and Swerling-I	256-sample subset spanning 1.023 s	SNR =−18 to −6 dB in 3-dB steps; Δa=4 m/s^2^	200 paired scenes per SNR; 6000/6000 per rule

Noise-window counts are reported as calibration/validation; the two sets are disjoint. Rule-specific thresholds target $P_{\mathrm{FA},\mathrm{gate}}=0.01$ and remain fixed across the SNR sweep.

## Data Availability

The original contributions presented in this study are included in the article/Supplementary Material. Further inquiries can be directed to the corresponding author.

## Supplementary Materials

The following supporting information can be downloaded at https://www.mdpi.com/article/10.3390/s26196121/s1, Figure S1: PRF and equivalent-reference-velocity operating-region sensitivity; Table S1: Sensitivity to mismatch in the equivalent cross-range reference velocity.
