# Supplementary material for: Robust Localization of Maneuvering UAV Swarms Using Lateral Doppler Resolution
Source: Sensors (Basel). 2026 Sep 26;26(19):6121. doi: 10.3390/s26196121 (PMC13667667; doi:10.3390/s26196121)
Supplement: Supplementary file 1 [file sensors-26-06121-s001.zip › sensors-4521353-supplementary.pdf]

Supplementary Materials: Robust Localization of Maneuvering UAV Swarms Using Lateral Doppler Resolution

Chenfei Yang, Zhuangzhi Han*, Jinwei Jia, Limin Liu, Lin Shi

S1. Operating-Region and Reference-Velocity Sensitivity

To quantify the operating conditions associated with the exact slant-range model, Monte Carlo experiments were conducted over the equivalent reference velocity and PRF. Each condition uses 200 independently randomized 15-target scenes at a mean per-pulse SNR of −15 dB, with Swerling-I scattering and complex Gaussian noise. Matched acceleration hypotheses are used so that cross-range observability and slow-time sampling can be examined separately. Each condition uses 3000 noise-only windows for threshold calibration and a disjoint set of 3000 windows for validation, with a target gate-level exceedance probability of 0.02. This operating point differs from the 0.01 target used in the main-text grid and SNR experiments, so the absolute detection probabilities are not intended as direct cross-experiment comparisons.

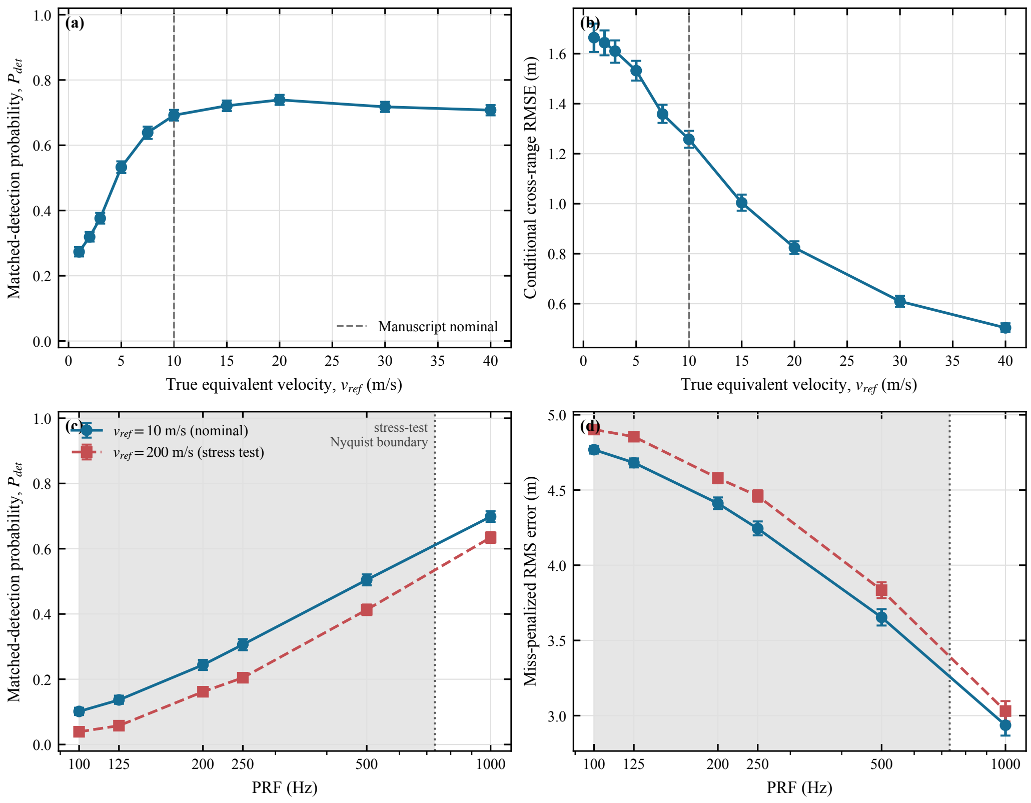

Figure S1. PRF and equivalent-reference-velocity operating-region sensitivity. Panels (**a**,**b**) show matched-detection probability and conditional cross-range RMSE (RMSE_x) versus the true equivalent reference velocity at 1 kHz. Panels (**c**,**d**) show matched-detection probability and miss-penalized RMS error versus PRF for the nominal 10 m/s condition and a separately defined 200 m/s stress condition. Error bars denote scene-level 95% bootstrap intervals. The PRF sweep keeps the CPI and per-pulse SNR fixed; consequently, the number of pulses changes with PRF.

At the nominal setting of $v_{\text{ref}} = 10$ m/s and PRF $= 1$ kHz, the normalized Doppler occupancy is 0.0107 and no target–pulse samples cross the Nyquist boundary. The separately defined high-velocity condition is used to expose the sampling transition: at 500 Hz, its occupancy is 1.462 and 17.4% of target–pulse samples cross the Nyquist boundary, whereas at 1 kHz the occupancy is 0.732 and the crossing fraction is zero. Because the CPI and per-pulse SNR are fixed, the PRF curves jointly reflect sampling, pulse count, and coherent integration.

Reference-velocity mismatch produces a progressive performance change around the nominal value, as summarized in Table S1. The matched value gives the highest detection probability and the lowest error among the tested assumptions; the $\pm 5\%$ cases retain probabilities of 0.588 and 0.651 compared with 0.696 at the matched value.

Table S1. Sensitivity to mismatch in the equivalent cross-range reference velocity.

δv_{ref} (%)	P_{det} (95% CI)	RMSE$_x$ (m)	Penalized RMS (m)
−20	0.192 (0.180–0.203)	1.717	4.558
−10	0.392 (0.375–0.408)	1.673	4.037
−5	0.588 (0.571–0.604)	1.501	3.411
0	0.696 (0.680–0.713)	1.239	2.943
+5	0.651 (0.635–0.667)	1.532	3.202
+10	0.489 (0.473–0.506)	1.724	3.771
+20	0.286 (0.273–0.299)	1.771	4.331

The true value is $v_{\text{ref}} = 10$ m/s. A matched detection requires a noise-calibrated threshold exceedance and a cross-range error no larger than 3 m. RMSE$_x$ is conditional on matched detections; the penalized RMS assigns 5 m to a missed or unassociated target. Confidence intervals use scene-level bootstrap resampling over 200 scenes.
